# Professional Advertising

**Published:** 1860-01

**Authors:** C. C. Dills


### PROFESSIONAL ADVERTISING.

BY C. C. DILLS.

"I remember a mass of things, but nothing distinctly: a quarrel, but nothing wherefore."

MESSRS. EDITORS:—In a recent article on "Dental Advertising *vs.* Dental Ethics," we protested against the tendency of some modern would-be dental ethics, to render unprofessional a more extended advertisement than "a simple announcement of the practitioner's name, title, and place of business." In doing this we confined ourselves to a few deductions from established rights and ethics—having bearing on the matter—deferring a consideration of it in its other bearing, till a time more expedient; meanwhile we have been pleased to note the continued agitation of such subjects as dental quackery, advertising, ethics, &c., &c.,—have been pleased to note the movement for "popular instruction,"—bearing with it the true principle of dental advertising—that of informing the uninformed of the importance and capabilities of Dentistry. And here we will be allowed to affirm, that we have *never* had any other view of advertising—however much we *may* have missed the *way;*—and when our more fastidious than discerning opponents would charge us with unprofessional or *selfish* advertising, and yet embrace the popular instruction method as *philanthropic*, we would expose their superficiality and show that the same "main spring of all human actions,"—the same *first law of our being—selfishness!* (not vulgar)—prompted alike their and our method;—we will be allowed to present these peculiar philanthropic motives, as mirrored by the *honest nature* of the *founder* of this new system of advertising. We quote—"This should all be free from any thing of an advertising character, and thus"—(or *that thus it may*)—"*have more weight*, and the more likely secure publication in the newspapers, as matter of general interest." Again—"Now all this done from *pure motives of good to the public*, uninfluenced by private interest or aggrandisement must prove beneficial to those by whom the writer is surrounded,—*and he be amply repaid eventually—as surely* as the farmer reaps where he sows."

"Consistency, thou art a jewel."

(The italicising is our own.)

Now we repeat, we are glad to see this advertising becoming "popular";—we hail it as the dawn of a new era in dentistry here in the west. Now no longer will quackery live and have its being,—feast and wax strong,—upon "popular ignorance." And now peace and quiet to our risibles, we will no longer be ordered to extract one tooth and "plant" another,—or asked if filling teeth really does do any good, or if we really can put in false teeth to do any good, or, "now do tell, how do you fas'en them in when there aint another tooth in the head? I'v hearn them say you bored holes in the jaw bone, but I believe I'd stump it awhile first." And now peace and quiet to our better natures, we will no longer be "painfully impressed with the ignorance and consequent neglect on the part of the farmers," (aye and the *townsmen*, the lawyer and the *physician*, the preacher and his charge,) "with regard to their teeth." And again, peace to our credit, and luxury to our households, we will no longer *realize* that the quacks make the money, "while gentlemen of worth and scholarship and skill are barely making a living."

*Surely*, "There's a good time coming."

But *seriously* would we advocate advertising, and seriously would we consider the questions, How, and *to what extent*, shall we advertise? To the first we answer: We approve of the new method, as far as it is practical and consistent with itself, and only recommend that it be *added to* whenever and wherever it may prove inefficient in accomplishing its ends: *profit to all.* We want no other advertising than *facts known;* this we *do* want, and—if we mistake not the marked inquietude of the "dignified silence" with which the youth and energy of our profession is wont to "wait for the world to come to them"—*we will have it.* The supercilious and such as can afford it, may raise the hue and cry of unprofessionality! may quack quackery and quacks! may play on their harp of a thousand strings" (the m-e-d-i-c-a-l profession!) and make asses of themselves, may *ignore* circumstances calling for more energy, effort, *advertising* to secure business than they put forth; but before they become *earnest* in the matter and would *legitimate* their refinement, we advise them to review their forces, to call a national exclusive convention, and form an association which shall discard *mechanical* dentistry as *unprofessional*, and enjoin secresy of all improvements and discoveries made, as furthering the interests of all concerned.

The world may ridicule the advertising of a Barnum or Bonner, a Brandriff or Ayres, a Townsend or Janes, but it will hang its head for shame, and blush at its own stupidity at last.

Life *is* "earnest." Business is a part of life, and *advertising* is its *index.*

To the second we answer: *advertise to the extent the case may call for.* If you have already advertised to the amount of twenty-five years' successful practice, or are located within conventionalities' fastidious sway, where alike vulgar advertising is superfluous, *desist from it; but allow your less fortunate brother to advertise the more.*

However much or little you may advertise, know you are right in quality, and *go ahead.*

Now as we dwell for a moment upon the propriety of a *liberal* advertising on the part of the profession generally, and thus come in direct conflict with views entertained by those in the profession, *to whom* we owe all for its present advanced state,—we must insist we do so with a feeling of deep respect—amounting in many instances to a personal reverence for such persons, and only justify our course at all on the ground that, backed by the paradox, leaders are always in extremes,—we are satisfied their position in these matters is not only ultra and impracticable, but in many cases *over-bearing.* And when you would talk of *over-selfishness*, we would charge upon this class a positive and insufferable over-selfishness; inasmuch as they seem to dictate general and special ethics, *solely from their own limited experience and circumstances.*

It is desirable to ascertain just here, wherein we agree with, and to what extent we differ from the more *elite* of our ethical law-givers. To do this we will consider some of their specifications separately. They assume, first, that advertising is professional. In this we agree. There is no diversity of opinion as to the propriety of certain kinds of advertising. But, say our law-givers, secondly, this advertising should be limited to an "announcemnnt of the practitioner's name, title, and place of business." Upon this specification we are at issue. Say the law-givers, then you would advocate publishing a "long list of specified prices, from the pulling of a tooth to the extraction of a fang, from inserting a simple plug to the more difficult cases of the art. You would follow the dictates of popularity rather than conscience, would win the way by means other than those of experience and merit." No gentlemen, not so! These resorts of quacks receive *our* anathemas no less than yours. Well, then "you would furnish the country with almanacs, inform the world of your prodigious talents through the medium of the newspaper; you would manufacture gas as well as teeth." By no means; we hold all such evidences of quackery, gentlemen, in no less contempt than you; they in no sense enlighten the public mind, or benefit the community. The advertising we advocate is a *public*, no less than a *professional necessity.* We would have it unostentatious and truthful; would have it contain just such matter, and in just such form, as would serve to bring the public mind up to an enlightened appreciation of our profession.

(Herein lies philanthropy; let us embrace it, and not ignore, or sneakingly receive, the reward ever attending such.)

Another class of opponents more liberal than the above, agree with us as to the necessity of *information given*—the policy of advertising—but, by a strong circumlocution, would advocate that the information be given through the country press by means of "essays," "free from any thing of an advertising character," and "from pure motives of good to the public," &c. To all of which we would further object but little. The means and medium employed, and the results aimed at are the same as in other advertising. We only prefer doing directly what these gentlemen propose doing indirectly, and we submit, whether by urging these essays upon the country press for gratuitous publication, a suspicion (if the truth) may not arise that the profession is merely grinding its own axe at the expense of the printer,—is spunging its way to competency and favor. Now, however desirable it may be to keep out of sight the fact, we are compelled to admit our belief that personal interest underlays all the professions. If it is desirable to have it understood that the dental profession is merely a benevolent association, it may be done by the entire abolition of fees, and in no other way.

Now we beg to be shown *wherein consists the unprofessionality of the method of advertising we advocate*—one differing from these we have cited as acknowledged professional, only in *efficiency!* What we are contending for here is just this: That there be an acknowledged professional advertising of

*gradations*, corresponding with the gradation of intelligence and wants of our respective localities. And, as collateral, that it be considered unprofessional—as it is illiberal—for any man, or set of men, to follow up a private opposition to any particular advertisement (coming within specified limits in *style*) by public expressions derogatory to the professional or private character of the author of such advertisement.

But we are reminded that we are a little fast here, are begging the question somewhat, and that our opponents are not dead, but ready for one more "master effort" against *all* advertising as such. We yield! and—"see how they run," —our law-givers turn out *en masse*, bringing with them the *medical profession*, and, combined, they produce a proof to a demonstration, a *syllogism: Quacks advertise;* quacks are unprofessional; *therefore*, advertising is unprofessional. Well, that's good! We retort:

1st, Indirectly. Rascals, villains, and the worst of men advertise in the business world. So do the best of men. Therefore the best of men are rascals, villains, and the worst of men.

2nd, Directly. Quacks advertise their "names, title, and place of business." So do professional men. Therefore, professional men are quacks.

But not to be foiled at their own game, they meet us again: *Professional* advertising is *limited*. Yours is not. *Therefore* yours is unprofessional.

Well, besides having destroyed this syllogism in having *limited* our advertising, we reply:

Unprofessional advertising is limitless. Ours *is* limited. Therefore ours *is professional*. But all such attempts to prove legitimate advertising unprofessional, reminds one of the unsuccessful attempt of a refined young man to obtain a *potatoe* of his neighbor at a dining table! Gent. "I say, stranger, can you reach those potatoes?" Stranger reaches forth his hand, finds he can, and replies "Yes." Gent, somewhat "sold," tries again, "Well, sir, just stick my fork in one of them!" Stranger does so, and leaves it there. Gent, ("sold again,") has him now, and says with an exultant air, "*Now sir, hand me my fork!*" Stranger deliberately does so, *leaving the potatoe.*

Still, many of our opponents (professionally weak kneed) linger, but to protest that we must not advertise so thoroughly, *because* our "mother profession" does not, *and we would court her favor.* Now here we would become *provoked!* would declare professional independence, self-reliance, *identity!* would want to know what we have to do with the *medical profession*, or it with us; or if a maternal "recognition" is insisted upon we want to know if *manifest merit* will not secure it more effectually than *ridiculous sycophancy.* We would want to know if the "eminently progressive and developmental character of our specialty" does not guarantee to it an everlasting *self-sustaining* endowment. And if it would not be well to "prove to the world that what is generally considered the only legitimate way to generate and develop the professional callings, is no more true than that all the bright children of earth have been begotten and born in legal wedlock"! Yes, we would want to know many things before giving this "protest" a serious consideration, but inasmuch as we remember that the number prostrate before this fatal Juggernaut is *fearful* we will be prevailed upon.

1st, Then, we would expostulate that our profession is so *peculiar*, we can not accept a precedent. (*Don't want one.*) 2nd, Our profession differs as widely from the medical profession as does *progress* from the law.

Now we will not say that we are not wholly and entirely *professional*, (that would be *heterodox*,) but we will say that there is nothing in the medical profession corresponding with our *mechanical dentistry*, which is ever and anon crying, "Progress, eternal progress! Reform! Excelsior!" and as earnestly proclaiming itself the "balm in Gilead" to "suffering humanity." If we have indeed a philanthropic chord in our souls, we would advertise our mechanical dentistry with its "very latest improvements," *faithfully*, and accept as our precedent the public benefactor any where. The dental profession is not to ignore the sufferings, ills and inconveniences, amounting in many instances to hazard of life and happiness, lying within its own peculiar sphere to alleviate, and a great auxiliary to which is proper advertising.

It *may* be more professional and humane for us to await in "dignified silence" "the world to come to us." Sluggishness *may* be an attribute of dignity, but if so, professional dignity is maintained at a fearful cost, both to the profession and the afflicted.

Conclusion: Our subject has been one worthy more experienced hands. But they have not—perhaps would never have—presented themselves. "Exciting causes" have prevailed with us. We are but just now realizing the extent and importance of our subject, the earnestness and sincerity of our endeavors. We have not written to encourage *quackery*, but to oppose *illiberality;* to foster *recklessness*, but the *reverse* in our ethics. Several important points we have merely alluded to, feeling that the familiarity of the profession with them, rendered detail—*of itself significant*—unnecessary. Some would have suggested a further handling of the matter "without gloves." By and by we may have the *manhood.*

